# Association Between Health-Related Quality of Life and Physical Functioning in Antiretroviral-Naive HIV-Infected Patients

**DOI:** 10.2174/1874613601812010117

**Published:** 2018-09-28

**Authors:** Ana Paula Lédo, Indira Rodriguez-Prieto, Liliane Lins, Mansueto Gomes Neto, Carlos Brites

**Affiliations:** 1 School of Medicine, Federal University of Bahia, Salvador, Bahia, Brazil; 2 Health Science Institute, Federal University of Bahia, Brazil; 3 Research Laboratory of Infectious Diseases, Edgard Santos Federal University Hospital, Salvador, Bahia, Brazil

**Keywords:** HIV infection, Acquired Immunodeficiency Syndrome, Health-related quality of life, HRQOL, Life Quality, Physical Conditioning Human

## Abstract

**Background:**

Poor functional status can significantly affect Health-Related Quality of Life (HRQoL) of HIV patients. However, there is scarce information on the functional profile of such patients before starting antiretroviral therapy (ART).

**Objective:**

To estimate the association between health-related quality of life and physical functioning in Antiretroviral-Naive HIV-infected patients.

**Methods:**

We conducted a cross-sectional study with HIV-infected patients older than 18 years, and naïve to antiretroviral therapy. The patients were evaluated for functional profile by pulmonary function (forced vital capacity, forced expiratory volume at one second, and Tiffeneau index), handgrip strength, and six-minute walk test in a cross-sectional study. HRQoL was evaluated by the 36-Item Short-Form Health Survey and its Physical (PCS) and Mental (MCS) Component Summaries. Multiple linear regression analyses were used to evaluate the association of predictor variables with PCS and MCS scores.

**Results:**

We found lower HRQoL among females patients, with far below average impairment of mental health component. Both male and female patients presented lower 6MWD function test values. Patients with dynapenia were older than patients without it, presented lower PCS mean score, lower family income, poor 6 MWD function test, lower FVC, and lower FEV1 t. Multivariable logistic regression analyses showed that Grip Strength, age and family income were predictor variables for Physical component of HRQoL. Female gender and smoking habit were predictive for the mental component of HRQoL.

**Conclusion:**

HRQoL in HIV, drug-naïve patients is predicted by level of dynapenia, smoking, income and gender. Therefore, lifestyle changes and active exercising can help to improve HRQoL in such patients.

## 1 INTRODUCTION:

HIV+/AIDS patients on successful **A**ntiretroviral Therapy (ART) present increased life expectancy. The use of better tolerated and efficacious antiretroviral drugs, improvement of adherence, prevention and proper management of non-communicable diseases, are the main reasons for the observed extension in patient’s survival [1]. Nevertheless, HIV-infected individuals still have a higher mortality risk when compared to the uninfected population, in consequence of non-AIDS related health problems [2]. On the other hand, the prevalence of HIV/AIDS is continually growing. Recent data revealed that 36.7 million people in the world are living with HIV and 18.2 million people are currently on antiretroviral therapy. In Brazil, approximately 830,000 individuals are infected with HIV, and 41,100 new cases are reported each year [3].

Although HIV infection/AIDS is currently considered a chronic disease it is still associated with disability, multi-factorial muscle wasting and physical impairment [4]. The physical changes resulting from HIV infection involve metabolic, neurological and muscular abnormalities [5], which may occur particularly in untreated patients. The use of ART has been associated with mitochondrial dysfunction and motor impairment. However, the occurrence of mitochondrial dysfunction in HIV untreated patients suggests that its involvement may be a non-ART related event [6].

HIV-infected people present decreased skeletal muscle mass that worsens as the disease progresses. Muscle wasting contributes to reduction of strength and functional performance due to the excess of protein degradation, induced lipodystrophy, and nutrient malabsorption [7]. Reduced muscle strength in HIV-infected patients may influence physical functioning and mental health, decreasing the performance of daily activities, and compromising patient´s health-related quality of life (HRQoL) [8].

HRQoL is a multidimensional concept that involves any changes in the health status, and considers physical, psychological, and social functioning aspects of the impact of a disease and/or a treatment on the individual HRQoL [9]. Several published studies reported poor HRQoL for HIV-infected patients [10-12], and functional impairment in those on ART, in association with low muscle mass and low bone mineral density [13].

To the best of our knowledge, only one study [14] has evaluated the correlation between functional profile and HRQoL of HIV+/AIDS patients during ART. Understanding the association between functional capacity and HRQoL in people with HIV+/AIDS before ART therapy may be useful for preventing functional decline and preserving patient’s physical and mental health. This study aims to estimate the association between health-related quality of life and physical functioning in antiretroviral-naive HIV-infected patients.

## 2 MATERIAL AND METHODS

### 2.1 Study Population

A cross-sectional study was conducted from February 2016 to October 2016. We invited consecutive, ART-naïve patients, aged ≥18 years, to participate in the study. Exclusion criteria included pregnancy, HIV-related clinical symptoms, and active opportunistic infections. Patients were recruited at the HIV Clinic of the Edgard Santos Federal University Hospital, Salvador, Bahia, Brazil.

### 2.2 Assessments

Background information included clinical history, demographic, socioeconomic and health-related characteristics. HIV-1 RNA plasma viral load and CD4/CD8-positive T-cells counts were recorded. Weight and height were measured, and the body mass index (BMI) was calculated. Functioning was evaluated by measuring pulmonary function, muscle strength, and functional capacity. Pulmonary function evaluation included standard measurements of forced vital capacity (FVC), forced expiratory volume at one second (FEV_1_) and Tiffeneau Index (FEV1/FVC). Patients were asked to take their maximal inspiration and then to expel air forcefully. A disposable mouthpiece and nose clip were used. The procedure was performed three times and the best result was recorded [15]. Handgrip strength was measured using a dynamometer. The dominant arm was positioned elbow and the participant was asked to squeeze the device as hard as possible for 3 seconds. The same procedure was repeated two times at intervals of 30 seconds, according to the protocol of the American Society of Hand Therapists [16]. The handgrip strength values were classified as expected or non-expected according to reference values of handgrip dynamometry of healthy adults obtained from a representative sample of adults living in Brazil in a population-based study. Dynapenia was defined as handgrip strength <30kg (men) and <20kg (women) [17]. The six-minute walk test was performed in a 30 meters-long straight corridor, with line-marks at every meter. Heart rate, peripheral oxygen saturation, blood pressure, and the Borg Scale were assessed at the second, fourth, and sixth minutes of the test [18]. The 6-MWD values were classified in expected and non-expected according to Iwama *et al.* [19] reference equation [6MWDm = 622.461 - (1.846 x Age years) + (61.503 x Sex (men = 1; women = 0].

Assessment of health-related quality of life was performed by using the SF-36 questionnaire (Medical Outcomes Study 36-Item Short-Form Health Survey). Eight scales (physical functioning, role limitations due to physical problems, bodily pain, general health perceptions, vitality, social functioning, role limitations due to emotional problems, and mental health) were aggregated into Physical Component Summary and Mental Component Summary scores. The eight scales scoring was performed using the Quality Metric Health Outcomes TM Scoring Software 4.0 to obtain the 0 to 100 algorithms and respective norm-based scores [20]. Normalized scores enable comparisons between the scales of the respective domain or component scale, transforming scores to a mean of 50 and standard deviation of 10. This study was licensed by Quality Metric Health Outcomes TM (number QM025905).

The Charlson comorbidity index (CCI) was calculated to access the severity of comorbid diseases. Scores were classified into three groups: mild (CCI scores of 1–2); moderate (CCI scores of 3–4); and severe (CCI scores ≥5) [21].

Data were analysed by using SPSS, version 22. We performed Shapiro-Wilk statistical tests to evaluate normality for relevant variables. Student's t-test was used to compare mean differences among sex and chi-square test was used to compare proportions.

## 3 RESULTS

The study group comprised a total of 104 patients with mean age of 34.99 ± 10.11 years, BMI of 23 ± 4.0 kg/m^2^, CD4 count of 403±284 cells/mm^3^ and CD8 count of 1180± 745.46 cells/mm^3^. The majority of them were male (76%) and black (81.8%); 48.1% had a family income lower than the Brazilian Minimal Wage (284.6 USD). Fifty-nine (56.7%) patients lived with their families and only 23.2% were engaged in a stable relationship. The majority of patients (74.1%) had nine or more schooling years.

The mean 6MWD for the entire population was 421.8 ± 102.3 m. Twenty females (80%) and 73 males (92.4%) have not accomplished the predicted values [18]. Considering the grip strength of dominant hand, 16 patients (15.4%) showed reduced muscle strength (Dynapenia). Respiratory disorders were evidenced in 36 (34%) of participants (low Tiffeneau Index). Patient characteristics are summarized in Table **1**.

In males, PCS was above average (52.8 ± 9.6), while MCS was below average (44.1 ± 12.7). Among the females, PCS was below average (45.5 ± 10.5), while mental health was far below average (35.2 ± 12.8). Females presented SF-36 scores significantly lower than males in all domains, except for role emotional (P = 0.118). The greatest differences between genders were Physical Functioning, Vitality, and Mental Health (P = 0.001), followed by Physical Component Summary (P = 0.002) and Mental Component Summary (P = 0.003).

Comparisons between measured FVC values and the FVC predicted values according to sex, showed that both males (4.5 ± 0.8 vs 3.6 ± 0.9, P < 0.001) and females (3.3 ± 0.5 vs 2.8 ± 0.8, P < 0.001) presented values significantly lower than the expected ones. Similar results were obtained for comparisons between FEV1 values and the FEV1 predicted values according to sex: both males (3.8 ± 0.6 vs 2.9 ± 0.8, P < 0.001) and females (2.8 ± 0.4 vs 2.3 ± 0.8, P = 0.003) presented values significantly lower than the expected ones. Again, comparisons between measured handgrip strength values and handgrip strength predicted values according to sex, showed that both males (42,8 ± 6.2 vs 39.2 ± 8.8, P = 0.003) and females (32.6 ± 8.7 vs 23.1 ± 7.0, P = 0.001) also showed values significantly lower than the expected ones. Mean handgrip strength of dominant hand was lower in females when compared to males, as expected (P < 0.001). Females covered a shorter distance than males 398.4 ± 78.2 m vs. 429.1 ± 108.1 m, but the difference was not statistically significant (*P* = 0.192). Comparisons between measured 6MWD values and 6MWD predicted values, using reference equation according to sex, showed that both males (429.1 ± 108.1 vs 620.9 ± 17.7, P < 0.001) and females (398.4 ± 78.2 vs 552.7 ± 20.6, P < 0.001) presented values significantly lower than the expected ones. Mean comparative analysis of SF-36 and functional tests by sex are shown in Table **2**.

Health-Related Quality of Life Summary Scores (PCS and MCS) stratified according to social demographic and clinical characteristics of the participants are shown in Table **3**.

Differences in Physical Component Summary were significant in family income (P = 0.025) and dynapenia (P = 0.001). The Mental Component Summary score was only associated with smoking habit (P = 0.041). Patients with less than nine years of schooling had lower scores when compared to individuals that had nine or more years of schooling, but it was not significant for both PCS (P=0.064) and MCS (P=0.711). However, the five illiterate patients had lower PCS and MCS mean scores: 39.3 ± 13.3 and 36.4 ± 15.3, respectively. Patients with dynapenia presented much lower PCS mean score (38.5 ± 11.7 *vs* 53.4 ± 8.2, P < 0.001), family income in USD (165.10 ± 186.1 *vs* 363.10 ±390.9, P=0.050), 6MWD (371.9 ± 110.1 *vs* 430.8 ± 98.7, P = 0.033), age (40.8 ± 9.8 vs 33.9 ± 9.8, P = 0.012), FVC (2.4 ± 0.6 vs 3.6 ± 0.9, P < 0.001)) and FEV1 (1.8 ± 0.4 vs 2.9 ± 0.8, P < 0.001) when compared with patients without dynapenia. The differences remained significant for patients younger (p = 0.02) or older than 40 years (p<0.001).

The association of the predictive variables with the PCS and MCS scores was explored using multiple regression analyses Table **4**. Age (P = 0.011), Family income (P =0.015), and Grip Strength (P<0.001) were strongly associated with the variation in Physical Component Summary; Gender (P = 0.008), and Smoking habit (P = 0.024) were good predictors of the Mental Component Summary.

## 4 DISCUSSION

This study showed lower HRQoL among HIV-treatment-naive female patients, with the mental health component mostly impaired and far below average. Both male and female HIV-infected patients presented lower than expected 6MWD function test values. Patients with dynapenia presented lower PCS mean score, lower family income, poor 6MWD function test, lower FVC, lower FEV1, but were older than patients without dynapenia. Multivariable logistic regression analyses showed that Grip Strength, age and family income were predictive variables for Physical component of HRQoL and female gender and smoking habit for the Mental component of HRQoL.

HIV infection and AIDS may result in substantial muscle wasting of multi-factorial origin and moderate combined resistance and aerobic training programs may improve strength, cardiac function, mood state and quality of life [4]. In our study, 80% of the females and 92.4% of the males have not accomplished the 6MWD test expected values. A cohort study [22] evaluated 354 patients, 90% on antiretroviral treatment in a two-year period. Function tests were impaired in HIV-infected adults when compared with published data from healthy individuals. Impaired physical function predicts disability and requires appropriate interventions to improve locomotor performance in HIV-infected patients. Diagnosis and routine use of locomotor test procedure, prior e during ART therapy, in HIV-infected patients are recommended.

Our study detected respiratory disorders (low Tiffeneau Index) in 34% of the individuals. Similarly, a cross-sectional study [23] evaluated respiratory disorders in 111 HIV-infected patients and 65 HIV-negative control matched by age, gender and smoking status. There was a higher prevalence of respiratory symptoms (P = 0.002), lower FEV1 (P = 0.002) and Tiffeneau index (P = 0.028) in HIV-infected than in HIV-negative controls. Authors recommended that HIV-infected patients should be screened for early diagnosis of respiratory disorders, preventing complications.

Low grip strength may be a marker of frailty and a risk factor for mortality among HIV patients [24]. In our study, 16 patients (15.4%) showed reduced muscle strength. A cohort study [25] investigated factors associated with grip strength in HIV patients, and evaluated nutritional, infectious and demographic factors. Multivariable analyses showed that poor grip strength was rather associated with nutritional than to infection or inflammation variables. The authors suggest the incorporation of regular functional evaluation and the use of grip strength as a functional indicator of nutritional improvement for HIV patients. In our study, patients with dynapenia presented lower family income (P = 0.050), lower 6MWD function test (P = 0.033), higher age (P = 0.012), lower FVC (P < 0.001) and FEV1 (P < 0.001) when compared with patients without dynapenia. Although the low family income could suggest a role for malnutrition in this population, the body mass index was not associated with tests performance. Older age usually is associated with dynapenia, but mean age of studied population was only 34 years. This suggests HIV infection play a role in promotion of Dynapenia for untreated patients.

Preventing complications and maximizing HRQoL in HIV-infected patients before ART therapy may contribute to effectiveness of treatment. Our study showed a gender-related difference in HRQoL. Mean PCS of male patients was above average (50%), while MCS was below average. Among women, both mean PCS and mean MCS were below average and the MCS was much lower than the PCS. Women presented lower SF-36 mean scores in all domains, when compared to men, except for role emotional (P = 0.118). The greatest differences between genders were in the physical domains (P = 0.001), although both physical (P = 0.002) and mental (P=0.003) Components Summaries also differed. These data are in accordance with the results of a randomized clinical trial [26] that evaluated HRQoL differences between men and women. At baseline, women had lower HRQoL scores than men in all of the domains except social functioning [26]. A recent study in our site detected a higher frequency of treatment failure in women, and a significant association of treatment failure and signs of depression and anxiety. Proper evaluation of HRQoL for women before starting ART could identify those with lower MCS and provide them interventions capable of improving HRQoL, especially in MCS, as prevention of treatment failure [27].

Our study evidenced that 6MWD function test was significantly much lower than the expected values (P<0.001), in men and in women. The multivariate analyses evidenced Grip Strength, age, and family income as predictive variables for PCS, a summary measure of physical component of the health-related quality of life. Previous reports show relevant correlations between decreased in physical function and in QoL with inadequate nutritional support and absence of adequate exercise programs in HIV-infected patients [28].

According to our results, gender (P = 0.008) and smoking (P = 0.024) were useful predictors of the mental component of the health-related quality of life. Our data are consonant with previous studies that evaluated the health-related quality of life in health subjects. A cross-sectional study [29] evaluated the HRQoL of 714 subjects without diagnosis of any physical or mental disorder; 44.7% were smokers and 55.3% non-smokers. Nicotine dependence was associated with significant impairment in the mental component summary (MCS) of the HRQoL. Several studies show a higher proportion of smokers in HIV-infected patients than in seronegative ones. Chronic smoking leads to decrease in respiratory function and increased risk of cardiovascular disease. In addition, smoking can be a marker of anxiety or depression, which is consistent with our results. Interventions to promote smoking cessation are mandatory in management of HIV patients [30, 31].

The cross-sectional design represents an important limitation in our study. We found important correlations, but it is not possible to establish causality, considering the predictive variables for HRQoL. However, the use of multivariate analysis showed a persistent association between some of study´s variables, and the main outcomes, minimizing the potential confounding factors. The lack of information on the determinants of HRQoL in Brazilian HIV patients also reinforces the need of studies in this field. To our knowledge, in Northeast Brazil, this is the first study to assess the functional profile and the HRQoL of people with HIV before starting ART. Prospective studies would be required, to define the role of ART and other interventions on HRQoL, in Brazilian HIV patients.

## CONCLUSION

Level of Dynapenia, age, and family income were useful predictors of the physical component of health-related quality of life in HIV drug-naïve patients, while gender and smoking were good predictors of the mental component of HRQoL. Therefore, lifestyle changes and active exercising can help to improve HRQoL in such patients.

## Figures and Tables

**Table 1:** Demographic and clinical characteristics of the 104 HIV-treatment-naive patients, Salvador, Bahia, 2017.

**Demographic and clinical characteristics**	**N**	**%**
**Gender**	-	-
Male	79	76
Female	25	24
**Educational status**	-	-
<9 years	21	18.8
≥ 9 years	83	74.1
**Marital status**	-	-
Single	78	69.6
Married/stable relationship	26	23.2
**Smoking status**	-	-
No	82	78.8
Yes	22	21.2
**Drinking**	-	-
No	95	91.3
Yes	9	8.7
**Body mass index (kg/m2)**	-	-
<25 – Underweight	12	11.5
25-29 - Normal weight	86	82.7
≥30 – Obese	6	5.8
**Living**	-	-
Alone	36	34.6
With Family	59	56.7
With Friends	9	8.7
**Skin color / Race/ Ethnicity**	-	-
African Brazilian	85	81.8
Caucasian	19	18.3
Family income (Minimal Wages)*	-	-
< 1 MW	50	48.1
≥ 1 MW	54	51.9
Charlson’s comorbidity index**	-	-
No	77	74
Mild	21	20.2
Moderate	5	4.8
Severe	1	1
**Functional tests**	-	-
6-Minute Walk Test Distance (m)***	-	-
< expected	93	89.4
≥ expected	11	10.6
Borg index****	-	-
Very light	33	31.7
Fairly light	68	65.2
Hard	3	2.9
Grip Strength (kg)*****	-	-
Dynapenia	16	15.4
Non- dynapenia	88	84.6
**Lung function**	-	-
Tiffeneau index (FEV1/FVC *100) < 80%	-	-
No	68	65.4
Yes	36	34.6

*Family income (Minimal Wages): 284.6 USD **Charlson’s comorbidity index: 0 - 6pontos. No: 0, mild: 1-2, moderate: 3-5, severe: 6 ***6-Minute Walk Test Distance (m): *Iwama et al*.^18^ equation [6MWDm = 622.461 - (1.846 x Age years) + (61.503 x Sex (men = 1; women = 0)] ****Borg index: Very light: 6 – 10; Fairly light: 11- 14; Hard: 15-20 *****Grip Strength (kg): Dynapenia: Based on handgrip strength <30kg (men) and <20kg (women)

**Table 2:** Means and standard deviations of health-related quality of life in 104 hIV-treatment-naive patients according to gender, salvador, bahia, 2017.

Health-Related Quality Life Domains and Functional Tests	Male (N=79)	Female (N=25)	P*
Physical Functioning (PF)	51.3 ± 9.4	40.1 ± 12.3	0.001
Role Physical (RP)	46.9 ± 11.9	40.1 ± 12.5	0.016
Bodily Pain (BP)	53.8 ± 11.9	46.1 ± 13.7	0.005
General Health (GH)	49.1 ± 9.9	42.9 ± 10.5	0.008
Vitality (VT)	52.9 ± 11.5	43.8 ± 10.9	0.001
Social Functioning (SF)	45.4 ± 11.3	38.5 ± 13.5	0.012
Role Emotional (RE)	41.9 ± 13.9	36.8 ± 14.3	0.118
Mental Health (MH)	46.8 ± 11.6	32.9 ± 14.0	0.001
Physical Component Summary (PCS)	52.8 ± 9.6	45.5 ± 10.5	0.002
Mental Component Summary (MCS)	44.1 ± 12.7	35.2 ± 12.8	0.003
Grip Strength (kg)**	39.2 ± 8.8	23.9 ± 6.9	0.001
6MWD***	429.1 ± 108.1	398.4 ± 78.2	0.192
Tiffeneau index*****	79.9 ± 15.0	85.5 ± 11.2	0.051

*Independent Samples Student-t Test ** Grip Strength (kg): Dynapenia: Based on handgrip strength <30kg (men) and <20kg (women) ***6-Minute Walk Test Distance (m) ****6-Minute Walk Test Distance (m): *Iwama et al*.^18^ equation [6MWDm = 622.461 - (1.846 x Age years) + (61.503 x Sex (men = 1; women = 0)] *****Tiffeneau index/ (FEV1/FVC x 100): Respiratory Disturbance: < 80%

**Table 3:** - Mean and Standard Deviation of PCS and MCS according to sociodemographic and clinical data in 104 HIV-treatment-naive patients, Salvador, Bahia, 2017.

-	**PCS** ^ז^	**P**	**MCS** ^זז^	P*
**Educational status**	-	-	-	-
<9 years (n=21)	47.3± 11.5	0.064	41.1 ± 13.9	0.711
≥ 9 years (n=83)	52.0 ± 13.8		42.2± 13.1	
**Smoking status**	-	-	-	-
No (n=82)	51.9 ± 10.4	0.995	43.3 ± 12.7	0.041
Yes (n=22)	51.1 ± 9.8		36.9 ± 14.1	
Family income (Minimal Wages)**	-	-	-	-
< MW (n=50)	48.7± 10.6	0.025	39.7± 13.7	0.094
≥ MW (n=54)	53.2 ± 9.5		44.0± 12.4	
**Marital status**	-	-	-	-
Alone (n=78)	51.3 ± 9.9	0.729	43.1 ±12.9	0.141
In couple (n=26)	50.5± 11.5		38.6± 13.8	
Grip Strength (kg)***	-	-	-	-
Dynapenia (n=16)	38.5 ± 11.7	0.001	39.7 ± 12.4	0.461
Non-dynapenia (n=88)	53.4± 8.2		42.4 ± 13.3	
6-Minute Walk Test Distance (m)****	-	-	-	-
< expected (n=11)	50.3 ± 9.1	0.800	39.7 ± 15.8	0.548
≥ expected (n=93)	51.2 ± 10.4		42.2 ± 12.9	
Tiffeneau index/ *****	-	-	-	-
No (n=73)	51.3 ± 9.6	0.741	40.8 ± 12.9	0.175
Yes (n=31)	50.6 ± 1.8		44.7 ± 13.6	

*Independent Samples Student-t Test ז PCS: Physical Component Summary; זז MCS: Mental Component Summary *Family income (Minimal Wages): MW: USD 285.00 **Grip Strength (kg): Dynapenia: Based on handgrip strength <30kg (men) and <20kg (women) ***6-Minute Walk Test Distance (m): Iwama *et al.*18 equation [6MWDm = 622.461 - (1.846 x Age years) + (61.503 x Sex (men = 1; women = 0)] ****Tiffeneau index/ (FEV1/FVC x 100): Respiratory Disturbance: < 80%

**Table 4:** Results obtained by applying a multiple regression equation having PCS and MCS as the dependent variable for 104 HIV-treatment-naive patients, Salvador, Bahia, 2017. The numbers in columns are regression coefficients (B), standard errors (SE_B_) and P values.

**PCS (R^2^ = 35%)**	**B**	**SE_B_**	P*
Constant	45.820	6.103	0.001
Gender (reference: female)	-1.516	2.675	0.572
Age, years	-0.236	0.091	0.011
Family income*	0.002	0.001	0.015
Grip Strength**	0.405	0.110	0.001
Body mass index***	-0.070	0.227	0.758
**MCS (R^2^ = 15%)**	**B**	**SE_B_**	P*
Constant	41.931	5.283	0.001
Gender (reference: female)	8.189	3.011	0.008
Age, years	-0.159	0.123	0.198
Family income*	0.001	0.001	0.459
Smoking habit	-6.929	3.013	0.024

*Family income (Minimal Wages): MW: USD 285.00 **Grip Strength (kg) ***Body mass index (kg/m2)
